# Supplementary material for: EnzML: multi-label prediction of enzyme classes using InterPro signatures
Source: BMC Bioinformatics. 2012 Apr 25;13:61. doi: 10.1186/1471-2105-13-61 (PMC3483700; doi:10.1186/1471-2105-13-61)
Supplement: Addtional file 5 — The Java code to format the data files, evaluate and predict. The file enzml_java_code.tar.gz contains the Java code used to format database data to ARFF and XML formats, to execute cross and train-test (jackknife) evaluations and to record evaluation results to database. More information is included in the readme.txt file and the Javadoc files. The code can be used with a MySQL database. To use a different database software, other JDBC drivers might be required. [file 1471-2105-13-61-S5.gz › java_code/utils/doc/uk/ac/ed/inf/utils/guiutils/GuiUtils.html]

GuiUtils


---


|  |  |  |
| --- | --- | --- |
| **Overview** \| **Package** \| **Class** \| **Use** \| **Tree** \| **Deprecated** \| **Index** \| **Help** | |  |
| **PREV CLASS**   **NEXT CLASS** | **FRAMES**    **NO FRAMES**     **All Classes** |  |
| SUMMARY: NESTED \| FIELD \| CONSTR \| METHOD | DETAIL: FIELD \| CONSTR \| METHOD |  |


---


## uk.ac.ed.inf.utils.guiutils Class GuiUtils

```
java.lang.Object
  java.awt.Component
      java.awt.Container
          java.awt.Window
              java.awt.Frame
                  javax.swing.JFrame
                      uk.ac.ed.inf.utils.guiutils.GuiUtils
```

**All Implemented Interfaces:**: java.awt.image.ImageObserver, java.awt.MenuContainer, java.io.Serializable, javax.accessibility.Accessible, javax.swing.RootPaneContainer, javax.swing.WindowConstants

---

``` public class GuiUtils extends javax.swing.JFrame ```

Gui utilities

**Version:**
:   10 Dec 2007

**Author:**
:   Luna De Ferrari luna.deferrari-at-ed.ac.uk

**See Also:**: Serialized Form

---

**Nested Class Summary**

**Nested classes/interfaces inherited from class java.awt.Component**

`java.awt.Component.BaselineResizeBehavior`


**Field Summary**

**Fields inherited from class javax.swing.JFrame**

`EXIT_ON_CLOSE`

**Fields inherited from class java.awt.Frame**

`CROSSHAIR_CURSOR, DEFAULT_CURSOR, E_RESIZE_CURSOR, HAND_CURSOR, ICONIFIED, MAXIMIZED_BOTH, MAXIMIZED_HORIZ, MAXIMIZED_VERT, MOVE_CURSOR, N_RESIZE_CURSOR, NE_RESIZE_CURSOR, NORMAL, NW_RESIZE_CURSOR, S_RESIZE_CURSOR, SE_RESIZE_CURSOR, SW_RESIZE_CURSOR, TEXT_CURSOR, W_RESIZE_CURSOR, WAIT_CURSOR`

**Fields inherited from class java.awt.Component**

`BOTTOM_ALIGNMENT, CENTER_ALIGNMENT, LEFT_ALIGNMENT, RIGHT_ALIGNMENT, TOP_ALIGNMENT`

**Fields inherited from interface javax.swing.WindowConstants**

`DISPOSE_ON_CLOSE, DO_NOTHING_ON_CLOSE, HIDE_ON_CLOSE`

**Fields inherited from interface java.awt.image.ImageObserver**

`ABORT, ALLBITS, ERROR, FRAMEBITS, HEIGHT, PROPERTIES, SOMEBITS, WIDTH`


**Constructor Summary**

`GuiUtils()`


| **Method Summary** | |
| --- | --- |
| `static void` | `errorPopUp(java.lang.String message)`             Error pop-up window with message (icon: stop) |
| `static java.io.File` | `fileChooserWithPreMessage(java.lang.String message, java.lang.String barTitle)`             1. |
| `static void` | `interactiveError(boolean isInteractive, java.lang.String message)`             Emits a popup if the mode is interactive or writes to System.err if non interactive |
| `static void` | `interactiveMessage(boolean isInteractive, java.lang.String message)`             Emits a popup if the mode is interactive or writes to System.out if non interactive |
| `static void` | `main(java.lang.String[] args)`             For testing |
| `static void` | `messagePopUp(java.lang.String message, java.lang.String barTitle)`             Pop-up window with message (icon: information > "i") |
| `static int` | `multiOptionsPopUp(java.lang.String message, java.lang.String title, java.lang.String[] options)`             A multiple options popup. |
| `static java.io.File` | `simpleDirectoryChooser()`             Returns the directory chosen by the user in a file chooser window. |
| `static java.io.File` | `simpleFileChooser()`             Returns the file chosen by the user in a file chooser window. |
| `static java.lang.String` | `simpleInputDialog(java.lang.String question, java.lang.String title)`             Creates a simple input pop-up |
| `static void` | `simpleMessagePopUp(java.lang.String message)`             Pop-up window with message (icon: information > "i") |
| `static int` | `yesNoPopup(java.lang.String message, java.lang.String title, java.lang.String option1, java.lang.String option2)`             A two options popup (usually for yes / no) The second option (usually the no) is pre-selected. |

**Methods inherited from class javax.swing.JFrame**

`getAccessibleContext, getContentPane, getDefaultCloseOperation, getGlassPane, getGraphics, getJMenuBar, getLayeredPane, getRootPane, getTransferHandler, isDefaultLookAndFeelDecorated, remove, repaint, setContentPane, setDefaultCloseOperation, setDefaultLookAndFeelDecorated, setGlassPane, setIconImage, setJMenuBar, setLayeredPane, setLayout, setTransferHandler, update`

**Methods inherited from class java.awt.Frame**

`addNotify, getCursorType, getExtendedState, getFrames, getIconImage, getMaximizedBounds, getMenuBar, getState, getTitle, isResizable, isUndecorated, remove, removeNotify, setCursor, setExtendedState, setMaximizedBounds, setMenuBar, setResizable, setState, setTitle, setUndecorated`

**Methods inherited from class java.awt.Window**

`addPropertyChangeListener, addPropertyChangeListener, addWindowFocusListener, addWindowListener, addWindowStateListener, applyResourceBundle, applyResourceBundle, createBufferStrategy, createBufferStrategy, dispose, getBufferStrategy, getFocusableWindowState, getFocusCycleRootAncestor, getFocusOwner, getFocusTraversalKeys, getGraphicsConfiguration, getIconImages, getInputContext, getListeners, getLocale, getModalExclusionType, getMostRecentFocusOwner, getOwnedWindows, getOwner, getOwnerlessWindows, getToolkit, getWarningString, getWindowFocusListeners, getWindowListeners, getWindows, getWindowStateListeners, hide, isActive, isAlwaysOnTop, isAlwaysOnTopSupported, isFocusableWindow, isFocusCycleRoot, isFocused, isLocationByPlatform, isShowing, pack, paint, postEvent, removeWindowFocusListener, removeWindowListener, removeWindowStateListener, reshape, setAlwaysOnTop, setBounds, setBounds, setCursor, setFocusableWindowState, setFocusCycleRoot, setIconImages, setLocationByPlatform, setLocationRelativeTo, setMinimumSize, setModalExclusionType, setSize, setSize, setVisible, show, toBack, toFront`

**Methods inherited from class java.awt.Container**

`add, add, add, add, add, addContainerListener, applyComponentOrientation, areFocusTraversalKeysSet, countComponents, deliverEvent, doLayout, findComponentAt, findComponentAt, getAlignmentX, getAlignmentY, getComponent, getComponentAt, getComponentAt, getComponentCount, getComponents, getComponentZOrder, getContainerListeners, getFocusTraversalPolicy, getInsets, getLayout, getMaximumSize, getMinimumSize, getMousePosition, getPreferredSize, insets, invalidate, isAncestorOf, isFocusCycleRoot, isFocusTraversalPolicyProvider, isFocusTraversalPolicySet, layout, list, list, locate, minimumSize, paintComponents, preferredSize, print, printComponents, remove, removeAll, removeContainerListener, setComponentZOrder, setFocusTraversalKeys, setFocusTraversalPolicy, setFocusTraversalPolicyProvider, setFont, transferFocusBackward, transferFocusDownCycle, validate`

**Methods inherited from class java.awt.Component**

`action, add, addComponentListener, addFocusListener, addHierarchyBoundsListener, addHierarchyListener, addInputMethodListener, addKeyListener, addMouseListener, addMouseMotionListener, addMouseWheelListener, bounds, checkImage, checkImage, contains, contains, createImage, createImage, createVolatileImage, createVolatileImage, disable, dispatchEvent, enable, enable, enableInputMethods, firePropertyChange, firePropertyChange, firePropertyChange, firePropertyChange, firePropertyChange, firePropertyChange, getBackground, getBaseline, getBaselineResizeBehavior, getBounds, getBounds, getColorModel, getComponentListeners, getComponentOrientation, getCursor, getDropTarget, getFocusListeners, getFocusTraversalKeysEnabled, getFont, getFontMetrics, getForeground, getHeight, getHierarchyBoundsListeners, getHierarchyListeners, getIgnoreRepaint, getInputMethodListeners, getInputMethodRequests, getKeyListeners, getLocation, getLocation, getLocationOnScreen, getMouseListeners, getMouseMotionListeners, getMousePosition, getMouseWheelListeners, getName, getParent, getPeer, getPropertyChangeListeners, getPropertyChangeListeners, getSize, getSize, getTreeLock, getWidth, getX, getY, gotFocus, handleEvent, hasFocus, imageUpdate, inside, isBackgroundSet, isCursorSet, isDisplayable, isDoubleBuffered, isEnabled, isFocusable, isFocusOwner, isFocusTraversable, isFontSet, isForegroundSet, isLightweight, isMaximumSizeSet, isMinimumSizeSet, isOpaque, isPreferredSizeSet, isValid, isVisible, keyDown, keyUp, list, list, list, location, lostFocus, mouseDown, mouseDrag, mouseEnter, mouseExit, mouseMove, mouseUp, move, nextFocus, paintAll, prepareImage, prepareImage, printAll, removeComponentListener, removeFocusListener, removeHierarchyBoundsListener, removeHierarchyListener, removeInputMethodListener, removeKeyListener, removeMouseListener, removeMouseMotionListener, removeMouseWheelListener, removePropertyChangeListener, removePropertyChangeListener, repaint, repaint, repaint, requestFocus, requestFocusInWindow, resize, resize, setBackground, setComponentOrientation, setDropTarget, setEnabled, setFocusable, setFocusTraversalKeysEnabled, setForeground, setIgnoreRepaint, setLocale, setLocation, setLocation, setMaximumSize, setName, setPreferredSize, show, size, toString, transferFocus, transferFocusUpCycle`

**Methods inherited from class java.lang.Object**

`equals, getClass, hashCode, notify, notifyAll, wait, wait, wait`

**Methods inherited from interface java.awt.MenuContainer**

`getFont, postEvent`

**Constructor Detail**

### GuiUtils

```
public GuiUtils()
```


**Method Detail**

### errorPopUp

```
public static void errorPopUp(java.lang.String message)
```

:   Error pop-up window with message (icon: stop)

    :   **Parameters:**: `message` -

---


### fileChooserWithPreMessage

```
public static java.io.File fileChooserWithPreMessage(java.lang.String message,
                                                     java.lang.String barTitle)
```

:   1. tells the user the reason she should select a file using a pop-up. 2.
    opens a file chooser windows. 3. Returns the file chosen by the user.

    :   **Returns:**: the file chosen by the user

---


### interactiveError

```
public static void interactiveError(boolean isInteractive,
                                    java.lang.String message)
```

:   Emits a popup if the mode is interactive or writes to System.err if non
    interactive

    :   **Parameters:**: `isInteractive` - true if the mode is interactive: `message` - the error message

---


### interactiveMessage

```
public static void interactiveMessage(boolean isInteractive,
                                      java.lang.String message)
```

:   Emits a popup if the mode is interactive or writes to System.out if non
    interactive

    :   **Parameters:**: `isInteractive` - true if the mode is interactive: `message` - the message

---


### main

```
public static void main(java.lang.String[] args)
```

:   For testing

    :   **Parameters:**: `args` -

---


### messagePopUp

```
public static void messagePopUp(java.lang.String message,
                                java.lang.String barTitle)
```

:   Pop-up window with message (icon: information > "i")

    :   **Parameters:**: `message` - the message in the pop-up: `barTitle` - the title for the bar above the pop-up

---


### multiOptionsPopUp

```
public static int multiOptionsPopUp(java.lang.String message,
                                    java.lang.String title,
                                    java.lang.String[] options)
```

:   A multiple options popup. The first option is pre-selected.

    :   **Parameters:**: `message` - the message / question: `title` - the title above the popup window frame: `options` - an array of string options **Returns:**: the int representing the chosen option (0 = first option, 1= second option ... -1 = the window was closed without selecting)

---


### simpleDirectoryChooser

```
public static java.io.File simpleDirectoryChooser()
```

:   Returns the directory chosen by the user in a file chooser window.

    :   **Returns:**: the chosen directory (as File object)

---


### simpleFileChooser

```
public static java.io.File simpleFileChooser()
```

:   Returns the file chosen by the user in a file chooser window.

    :   **Returns:**: the file chosen

---


### simpleInputDialog

```
public static java.lang.String simpleInputDialog(java.lang.String question,
                                                 java.lang.String title)
```

:   Creates a simple input pop-up

    :   **Parameters:**: `question` - the question: `title` - the pop-up bar title **Returns:**: the user input

---


### simpleMessagePopUp

```
public static void simpleMessagePopUp(java.lang.String message)
```

:   Pop-up window with message (icon: information > "i")

    :   **Parameters:**: `message` - the message in the pop-up

---


### yesNoPopup

```
public static int yesNoPopup(java.lang.String message,
                             java.lang.String title,
                             java.lang.String option1,
                             java.lang.String option2)
```

:   A two options popup (usually for yes / no) The second option (usually the
    no) is pre-selected.

    :   **Parameters:**: `message` - the message / question: `title` - the title above the popup window frame: `option1` - the option "To be...": `option2` - the other option "Not to be..." **Returns:**: the int representing the chosen option (0 = first option, 1= second option ... -1 = the window was closed without selecting)


---


|  |  |  |
| --- | --- | --- |
| **Overview** \| **Package** \| **Class** \| **Use** \| **Tree** \| **Deprecated** \| **Index** \| **Help** | |  |
| **PREV CLASS**   **NEXT CLASS** | **FRAMES**    **NO FRAMES**     **All Classes** |  |
| SUMMARY: NESTED \| FIELD \| CONSTR \| METHOD | DETAIL: FIELD \| CONSTR \| METHOD |  |


---
